# Chemical state mapping of simulant Chernobyl lava-like fuel containing material using micro-focused synchrotron X-ray spectroscopy

**DOI:** 10.1107/S1600577521007748

**Published:** 2021-09-13

**Authors:** Hao Ding, Malin C. Dixon Wilkins, Lucy M. Mottram, Lewis R. Blackburn, Daniel Grolimund, Ryan Tappero, Sarah L. Nicholas, Shikuan Sun, Claire L. Corkhill, Neil C. Hyatt

**Affiliations:** a NucleUS Immobilisation Science Laboratory, Department of Materials Science and Engineering, University of Sheffield, Sheffield S1 3JD, United Kingdom; b Swiss Light Source, Paul Scherrer Institute, Villigen, Switzerland; c Brookhaven National Laboratory, NSLS-II, Upton, NY 11973, USA; d School of Material Science and Energy Engineering, Foshan University, Foshan, Guangdong 528000, China

**Keywords:** micro-XAS, micro-XRF, micro-XRD, speciation map, multimodal

## Abstract

Uranium speciation and redox behaviour is of critical importance in the nuclear fuel cycle. X-ray absorption near-edge spectroscopy (XANES) is commonly used to probe the oxidation state and speciation of uranium, and other elements, at the macroscopic and microscopic scale, within nuclear materials. Two-dimensional (2D) speciation maps, derived from microfocus X-ray fluorescence and XANES data, provide essential information on the spatial variation and gradients of the oxidation state of redox active elements such as uranium. In the present work, we elaborate and evaluate approaches to the construction of 2D speciation maps, in an effort to maximize sensitivity to the U oxidation state at the U *L*_3_-edge, applied to a suite of synthetic Chernobyl lava specimens. Our analysis shows that calibration of speciation maps can be improved by determination of the normalized X-ray absorption at excitation energies selected to maximize oxidation state contrast. The maps are calibrated to the normalized absorption of U *L*_3_ XANES spectra of relevant reference compounds, modelled using a combination of arctangent and pseudo-Voigt functions (to represent the photoelectric absorption and multiple-scattering contributions). We validate this approach by microfocus X-ray diffraction and XANES analysis of points of interest, which afford average U oxidation states in excellent agreement with those estimated from the chemical state maps. This simple and easy-to-implement approach is general and transferrable, and will assist in the future analysis of real lava-like fuel-containing materials to understand their environmental degradation, which is a source of radioactive dust production within the Chernobyl shelter.

## Abstract 2

This work elaborates and evaluates approaches to the construction of 2D speciation maps, in an effort to maximize sensitivity to the U oxidation state at the U *L*_3_-edge, applied to a suite of synthetic Chernobyl lava specimens.

## 1. Introduction

Understanding uranium redox behaviour in materials generated in the nuclear fuel cycle and, importantly, those formed during nuclear accidents, is essential when developing effective remediation strategies to mitigate the impact of this radioactive element in the environment. U predominantly presents as poorly mobile, insoluble U^4+^ under reducing conditions, for example, in the sub-surface. In contrast, U^6+^ is prevalent in oxidizing conditions, with a higher solubility, for example in oxic groundwater. Nuclear fuel, predominantly UO_2_, exhibits extremely low corrosion rates in groundwater under anoxic conditions; however, the corrosion rate increases significantly when oxidative corrosion occurs, *i.e.* oxidation of U^4+^ to U^6+^ by oxidative and radiolytic solution species (Shoesmith, 2000 ▸). Likewise, it has been shown that borosilicate glass immobilizing U_3_O_8_ exhibited leaching rates in oxic ground water that were four times higher than in anoxic groundwater, due to the rapid oxidation and release of U (Jantzen & Trivelpiece, 2017 ▸). Crucially, the oxidation state of U within the material itself has also been found to influence corrosion rates; for borosilicate and aluminosilicate glasses immobilizing U, those with a higher mean oxidation state demonstrated the greatest extent of U release (Barlow *et al.*, 2021 ▸).

For the safe management of severely damaged nuclear fuel-containing materials such as Chernobyl Lava-like Fuel Containing Materials (LFCM) and Fukushima Molten Core Concrete Interaction products (MCCI) which, while remaining within the damaged reactor units, are a significant source term of mobile radioactivity, it is essential to develop techniques and skills to obtain an understanding of the uranium oxidation states and their evolution under alteration. For example, it is known that corrosion of LFCMs has occurred within the Chernobyl shelter, presumably by oxidative processes, leading to U dissolution, migration and secondary phase formation (Teterin *et al.*, 1994 ▸; Krinitsyn & Shcherbin, 2003 ▸; Burakov *et al.*, 1996 ▸; Baryakhtar *et al.*, 1997 ▸; Badovskii *et al.*, 2014 ▸). Elucidation of the underpinning mechanism for this phenomenon, which is a major source term for the production of haza­rdous radioactive dust, requires an understanding of the U oxidation state both within the LFCM and the corrosion products. Moreover, LFCMs can be generally classified as glass ceramic composites comprising multiple types of crystalline material, with U segregated in almost all of the phases, to a differing extent, with potentially different speciation. Due to the highly heterogeneous nature of these materials (Anderson *et al.*, 1992 ▸; Geisler *et al.*, 2006 ▸; Borovoi *et al.*, 1990 ▸; Savonenkov *et al.*, 1991 ▸; Shiryaev *et al.*, 2016 ▸; Burakov *et al.*, 1997 ▸), it is therefore important to be able to characterize the oxidation state of U at the microscale and with spatial resolution so as to understand their degradation, for future retrieval. Within the framework of an IAEA Co-ordinated Research Programme, our motivation is to develop and validate the necessary multimodal spectroscopy methods and analysis tools to support future analysis of real high-dose-rate Chernobyl LFCM and Fukushima MCCI materials.

X-ray absorption near-edge spectroscopy (XANES) in the hard X-ray regime has been widely used to investigate U speciation at the *L*_3_-edge. Much of the previous work shows that the oxidation states of U compounds follow an approximate linear relationship with the white line position in XANES spectra [the strong absorption feature(s) associated with the crest of the X-ray absorption edge]. This is because the effective nuclear charge, and thus the minimum energy of excitation, increases as the oxidation state increases (Bès *et al.*, 2016 ▸; Hunter & Bertsch, 1998 ▸). Investigations of U compounds and glasses, with a range of different structures, have illustrated that the profile of the white line and near-edge structure are also sensitive to the local environment. In particular, a post-edge feature observed at approximately 15 eV above the white line has been shown to be characteristic of the uranyl moiety (the linear UO_2_^2+^ oxocation), and attributed to ‘resonant’ multiple scattering of the photoelectron by the short uranyl U=O double bonds (Farges *et al.*, 1992 ▸; Den Auwer *et al.*, 2003 ▸). Asymmetry of the white line peak, with a shoulder at lower energy, was also reported to be an intrinsic feature of ternary oxide U compounds with oxidation state U^5+^ (Soldatov *et al.*, 2007 ▸). These changes in shape of the spectral envelope, as well as the occurrence of specific features, are in good agreement with corresponding calculations of the density of states and full multiple-scattering calculations (Shundalau & Umreiko, 2014 ▸; Yun *et al.*, 2007 ▸; Infante *et al.*, 2007 ▸; Hudson *et al.*, 1995 ▸; Den Auwer *et al.*, 2004 ▸). Differences in the U local environment, such as coordination number, symmetry, ligand types and distance, affect the hybridization and energy splitting of 5*f* and 6*d* orbitals, and, therefore, change the energy of the final state and the necessary excitation energy (Denning *et al.*, 2002 ▸; Den Auwer *et al.*, 2003 ▸; Bès *et al.*, 2016 ▸). For example, the covalent nature of U–ligand bonds was found to be greater with higher oxidation states and the further splitting of the available energy levels resulted in broadening of XANES features (Vitova *et al.*, 2017 ▸; Bès *et al.*, 2016 ▸; Bagus *et al.*, 2017 ▸). An example of the effect of the cluster geometry is seen in CaUO_4_ (Bagus *et al.*, 2017 ▸; Bès *et al.*, 2016 ▸); although this compound does contain uranyl moieties, the uranyl (axial) bonds are particularly long, and, consequently, the characteristic post-edge uranyl feature is ill defined (Barlow *et al.*, 2017 ▸, 2020 ▸; King, 2002 ▸). Even though these effects have been well documented, it remains a challenge to quantify these effects on the overall envelope of XANES spectra. Therefore, to extract and estimate the oxidation state accurately, linear combination methods of the XANES spectra generally require the use of reference spectra from U compounds or glasses with identical or very similar U coordination environment (Kosog *et al.*, 2012 ▸; Szymanski & Scott, 1982 ▸; Farges *et al.*, 1992 ▸).

To develop a quantitative and spatially resolved understanding of the U oxidation states present in heterogeneous environmental specimens, a chemical state mapping method based on XANES spectra has been applied, exploiting microscale X-ray fluorescence (XRF) maps at multiple energies over the U *L*_3_ absorption edge (Crean *et al.*, 2014 ▸). Chemical state maps were constructed at the edge energy and post-edge energy, normalized, and a linear function was applied to estimate the average local U oxidation state of depleted uranium particles (Crean *et al.*, 2014 ▸). U in these particles was mainly in the form of U oxide phases, with variations in the U/O stoichiometry. Such similar chemical environments allow a precise mapping of quantitatively determined oxidation states, because the oxidation state and coordination number depend only on the stoichiometry of this system. However, hitherto, there is limited consideration of the application of such a method to materials composed of multiple complex uranium-bearing phases. Nevertheless, methods have been developed for chemical state mapping of complex iron-bearing phase assemblages, based on linear fitting of Fe *K*-edge XANES and application of principle component analysis. However, they are considered as broadly qualitative analyses because the contributions of oxidation state and coordination number cannot be adequately deconvoluted (Lam *et al.*, 2012 ▸; Mayhew *et al.*, 2011 ▸). It has been suggested that quantitative maps can only be produced for systems containing only a single crystalline or amorphous phase (such as garnets) (Berry *et al.*, 2013 ▸), or for more complex systems by using multivariate statistical models, such as partial least squares (PLS), to determine the interrelationships between channels (energies) (Dyar, Breves *et al.*, 2016 ▸; Dyar, McCanta *et al.*, 2016 ▸).

In this contribution, we describe the development of methods to quantitatively estimate U oxidation states, in micrometre-sized pixels, within µ-XRF maps collected at multiple energies over the U *L*_3_ absorption edge. For the purpose of method development, we apply these methods to investigate representative low-activity simulant Chernobyl LFCMs, that closely approximate the composition and microstructure of the core melt down product formed in the Chernobyl nuclear accident (Kiselev & Checherov, 2001 ▸; Ushakov *et al.*, 1996 ▸; Burakov *et al.*, 1997 ▸; Borovoi *et al.*, 1998 ▸), but without the inclusion of short-lived fission product nuclides (Barlow *et al.*, 2017 ▸, 2020 ▸; Ding *et al.*, 2021 ▸). Previous investigation of simulant LFCMs by analysis of bulk U *L*_3_ XANES demonstrated a narrow range of average oxidation states of 4.0–4.5+ (Barlow *et al.*, 2017 ▸, 2020 ▸). Here, we demonstrate and validate refined µ-XRF mapping methods which accurately estimate the spatial distribution of average oxidation states in these complex materials.

## 2. Experimental methods

### 2.1. Materials

The preparation, synthesis and bulk characterization of simulant Chernobyl Brown and Black LFCM materials was performed as follows. The batched compositions were based on an average of all of the analysed real LFCM samples available in the literature [see Barlow *et al.* (2020 ▸) for a summary and for the final batch compositions]. Stoichiometric amounts of the precursors including SiO_2_ (Lochaline Quartz Sand, 99.6%), CaCO_3_ (Fisher, 98%), ZrO_2_ (Aldrich, 99%), Na_2_CO_3_ (Alfa Aesar, 98%), BaCO_3_ (Alfa Aesar, 99%), Al(OH)_3_ (Acros, 95%), Mn_2_O_3_ (Aldrich, 99%), stainless steel 316 (Fe/Cr_18_/Ni_10_/Mo_3_, Goodfellow), Mg(OH)_2_ (Sigma-Aldrich, 99.9%) and UO_2_ (BDH) were mixed and then heated in alumina crucibles under a reducing atmosphere (5% H_2_ in 95% N_2_) at 1500°C for 4 h, followed by a second dwell at 720°C for 72 h to encourage crystallite growth. Bulk characterization was conducted by powder X-ray diffraction (XRD) and scanning electron microscopy (SEM), demonstrating that the microstructures and phase assemblages of the simulant samples were similar to those found in real Brown and Black LFCMs (Barlow *et al.*, 2017 ▸, 2020 ▸).

### 2.2. Multimodal micro-focus X-ray analysis.

Multimodal micro-focus X-ray absorption spectroscopy and XRD measurements were conducted at the National Synchrotron Light Source II (NSLS-II), Brookhaven National Laboratory, USA, on beamline 4BM (XFM). Samples were prepared for µ-XRF, µ-XANES and µ-XRD analysis by mounting on 250 µm-thick Spectrosil fused quartz slides. Mounted samples were thinned and polished to a final approximate thickness of 50 µm by standard metallographic procedures. The XFM beamline utilizes a fixed-exit double-crystal monochromator [a pair of Si (111) crystals] for a consistent beam offset (25 mm) and a broad energy range (2.05–23 keV). A Kirkpatrick–Baez (KB) mirror system was utilized to focus the X-ray beam; the spot size of the beam was initially set as 10 µm × 10 µm for mapping of large areas of interest and then focused to 1.5 µm × 1.5 µm for acquisition of high-resolution µ-XRF maps. Samples were mounted on a motorized three-axis sample stage, positioned at 45° to the incident beam, behind the KB mirror. A Canberra 13-element Ge array detector, positioned at 90° to the incident beam, was used to measure the XRF emissions.

Prior to measurement, X-ray energy was calibrated using the *K*-edge of a standard yttrium foil (17038.4 eV). µ-XRF maps were collected at five selected energies in fly scanning mode, with a step size of 1.5 µm and a dwell time of 200 ms pixel^−1^. The individual map was set as 150 µm × 150 µm in size with 10000 pixels and the entire acquisition time was about 40 min. The energies were selected based on the XANES spectra of U oxometallate reference compounds (see detailed discussion below) including UO_2_ [U oxidation state (OS) 4+ and coordination number (CN) 8]; UO_3_ (OS 6+ and CN 6); CaUO_4_ (OS 6+ and CN 8); LaUO_4_ (OS 5+ and CN 8); UTiO_5_ (OS 6+ and CN 7); UTi_2_O_6_ (OS 4+ and CN 6); and Ca_3_UO_6_ (OS 6+ and CN 6). All these reference compounds were prepared by distributing ceramic powders into polyethyl­ene glycol (PEG) as a pellet and were confirmed as single phase by X-ray diffraction pattern.

Regions of interest were first determined by observing and processing the XRF images using the software *GSE Mapviewer* from Larch (Newville, 2013 ▸). U *L*_3_-edge µ-XANES spectra of the selected points were collected in fluorescence mode over the energy range 17050–17500 eV, with a resolution of 0.25 eV. Three individual XANES scans of each point of interest were collected, normalized and merged using *Athena* (Ravel & Newville, 2005 ▸). The average oxidation state at each point was estimated by using a linear regression of the threshold energy (*E*_0_) against the known oxidation state of reference compounds, and by linear combination fitting using the XANES data of reference compounds. The linear regression method uses the threshold energy (first inflection point in this paper) to represent the excitation energy, which is assumed to be a linear function of the U oxidation state. The linear combination fitting method used the combination of XANES spectra of reference compounds to fit the XANES spectrum of an unknown sample directly; the mean oxidation state of the unknown was determined from the weighted combination of reference compounds. Micro-focus extended X-ray absorption fine-structure (µ-EXAFS) data were normalized and a Fourier transform applied over the *k*-range 3.0–11.0 Å^−1^ using *Athena* and *Artemis*, parts of the *Demeter* software package (Rehr *et al.*, 2010 ▸). Scattering paths were calculated by employing the *FEFF* code as implemented in *Artemis*, including amplitude, phase shift, mean free path and the initial path lengths. The fitting was conducted by fixing the amplitude reduction factor at 0.95, using the same Debye−Waller factors for all paths that comprised the first shell of oxygen ions, and simultaneously refining the inter­atomic distances and coordination numbers. µ-XRD patterns were collected simultaneously over a range of 5–35° 2θ, with a resolution of 0.012°. Azimuthal integration of individual 2D µ-XRD patterns was performed using the *Dioptas* software package (Prescher & Prakapenka, 2015 ▸). The phase assemblage was determined by matching the reflections observed in the XRD patterns with materials previously reported in the ICSD and and ICDD databases.

### 2.3. Deconvolution of U *L*_3_-edge XANES spectra

The U *L*_3_-edge primarily corresponds to the excitation of core electrons from 2*p*_3/2_ states to 6*d* states, by absorption of X-ray photons. According to the first-order approximation of Fermi’s Golden rule (Rehr & Ankudinov, 2005 ▸), the probability of transition from initial states *i* (2*p*_3/2_) to final states *f* (6*d*), associated with the U *L*_3_-edge, is written according to equation (1),

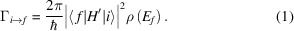

Here, *f*|*H*′|*i* is the matrix element of all possible perturbations, including the Auger effect and multiple scattering, from the initial state to final state, and ρ(*E*_*f*_) is the density of final states. Assuming the final states have discrete energies, the transition probability can be simplified as

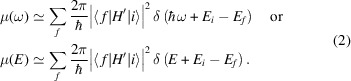

Here, ω represents the incident X-ray angular frequency, ℏω or *E* is the photon energy, and *E*_*i*_ and *E*_*f*_ are the initial and final state energy, respectively.

**Figure 1:**
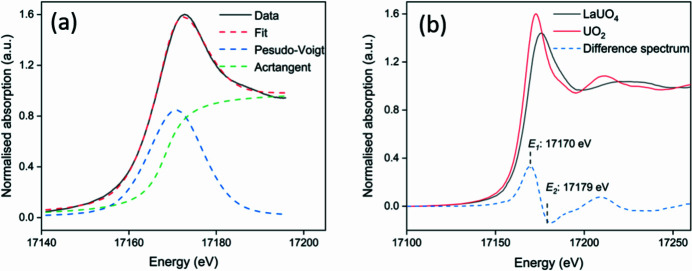
(*a*) Deconvolution of the XANES spectrum of UO_2_ reference compound into an edge step modelled by an arctangent function and pseudo-Voigt function to model the multiple-scattering component; (*b*) UO_2_ and LaUO_4_ XANES spectra and their difference spectrum. XRF maps at two energies (*E*_1_: 17170 eV; *E*_2_: 17179 eV) corresponding to maximum contrast between the XANES spectra were collected for chemical state map construction.

In simple terms, the XANES may be considered as arising from two key distinctive processes (Den Auwer *et al.*, 2003 ▸), which are summarized and represented as a matrix element *f*|*H*′|*i* in equation (1): (i) at low energies, the excited initial state electrons (2*p*) are primarily trapped into vacancies within the final states (6*d*); and (ii) at higher energies, a photoelectron is generated which is strongly scattered by neighbouring atoms in a multiple-scattering process. The scattering paths and probabilities are dependent on the chemical environment of the absorbing atom, defined by the number, type and distance of neighbouring atoms, and the point symmetry of the environment (Hudson *et al.*, 1995 ▸). Therefore, the U *L*_3_ XANES spectrum can be simply deconvoluted using two components: an edge step modelled using an arctangent function and a pseudo-Voigt function to model the multiple-scattering component. This is illustrated for UO_2_ in Fig. 1 ▸(*a*).

### 2.4. Chemical state mapping

#### 2.4.1. Consideration of reference compounds

XANES spectra can provide information on the average oxidation state within a material, since the oxidation state primarily determines the effective nuclear charge and the energy of the final states. Based on preliminary analysis of the U *L*_3_ µ-XANES spectra of points of interest in the simulant LFCMs, the threshold energies, *E*_0_, were determined to be between those of the UO_2_ (U^4+^) and LaUO_4_ (U^5+^) reference compounds, and not consistent with U^6+^ reference compounds. In addition, the µ-XANES spectra were not observed to present a post-edge resonance or shoulder above the white line attributed to the multiple scattering associated with the uranyl UO_2_^2+^ oxocation (Den Auwer *et al.*, 2003 ▸). This is consistent with previous bulk XRD and SEM/EDX analysis (Barlow *et al.*, 2020 ▸), and µ-XRD analysis of the points of interest and the surrounding region, which showed the formation of UO_2_, (U,Zr)O_2_ solid solution, and (U,Zr)SiO_4_ phases. In common with the selected reference compounds, for which µ-XANES and corresponding difference spectrum are shown in Fig. 1 ▸(*b*), these contain UO_8_ polyhedra (Ding *et al.*, 2021 ▸).

#### 2.4.2. Energy selection for XRF mapping

Given the µ-XANES difference spectrum of UO_2_ and LaUO_4_ reference compounds [Fig. 1 ▸(*b*)], two energies (*E*_1_: 17170 eV; *E*_2_: 17179 eV) corresponding to maximum contrast between the XANES spectra were chosen for µ-XRF mapping, corresponding to the greatest difference of normalized absorption between U^4+^ and U^5+^. Besides these two energies, a pre-edge energy position (*E*_*b*_: 17030 eV) for background subtraction and two post-edge energy positions (*E*_*p*1_: 17500 eV; *E*_*p*2_: 18000 eV) for normalization were also selected. Collection of µ-XRF maps at these energies was to ensure the conformity of the normalization between µ-XRF intensity and µ-XANES absorption coefficient. As the µ-XRF intensity (*I*_*f*_) is correlated to the µ-XANES absorption coefficient [μ(*E*)] in fluorescence mode [see equation (3)] with a fixed incident intensity (*I*_0_), the contrast in a normalized µ-XANES absorption spectrum is proportional to the corresponding contrast in normalized µ-XRF intensity,

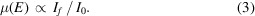

Edge step normalization was performed on the µ-XANES spectra of reference compounds using *Athena* (Ravel & Newville, 2005 ▸). The pre-edge line, post-edge line and normalization constant [μ_0_(*E*_0_)] are the three components controlling the normalization. The pre-edge line [*b*(*E*)] was regressed to the data in the pre-edge range and subtracted from μ(*E*) over all energies. The subtracted μ(*E*) was then divided by the normalization constant [μ_0_(*E*_0_)]. The evaluation of μ_0_(*E*_0_) for a µ-XANES spectrum is to extrapolate the pre- and post-edge lines to *E*_0_, and subtract the *E*_0_-crossing of the pre-edge line from the *E*_0_-crossing of the post-edge line [*p*(*E*_0_) − *b*(*E*_0_)]. The post-edge line [*p*(*E*)] is a three-term quadratic polynomial regressed to the data over the normalized range. The normalized absorption [μ(*E*)′] could be written as

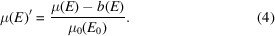

For normalization of µ-XRF intensity, the order of magnitude of the µ-XRF intensity in the pre-edge region is less than that at *E*_1_, *E*_2_, and in the post-edge region, based on preliminary observation; the pre-edge line was assigned to be a constant value (*I*_*b*_), collected from *E*_*b*_. The post-edge line was determined by a linear function [ 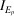 = 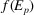 ] using the XRF intensities (*I*_*p*1_ and *I*_*p*2_) collected at energies *E*_*p*1_ and *E*_*p*2_. This was demonstrated to be more accurate compared with the conventional approach of assigning a single post-edge µ-XRF intensity, as the µ-XRF intensity over the post-edge range is not constant. The normalization constant could then be evaluated by subtracting *I*_*b*_ from the intensity [*f*(*E*_0_)] at *E*_0_ of the post-edge line. Therefore, the normalization of µ-XRF intensity at each pixel of the maps collected at *E*_1_ and *E*_2_ could be performed in the same way to the µ-XANES spectrum,

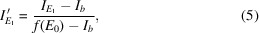




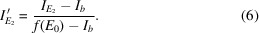

Here, 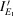 and 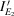 are the normalized µ-XRF intensities at *E*_1_ and *E*_2_ for any pixel from the map.

### 2.5. Data processing methods

#### 2.5.1. Gradient linear function (GLF) analysis

The difference spectrum derived from the U *L*_3_ µ-XANES spectra of UO_2_ and LaUO_4_ reference compounds [Fig. 1 ▸(*b*)] shows an almost linear dependence of the normalized absorption between *E*_1_ and *E*_2_. As the excitation energy is strongly dependent on the oxidation state(s) present, then at any particular map pixel the average oxidation state can be plausibly assumed to be a linear function of the difference in normalized µ-XRF intensities measured at *E*_1_ and *E*_2_. By tuning the incident energy to the five selected energies, µ-XRF maps were collected and normalized to estimate the average U oxidation state at each pixel and reveal the spatial distribution. The estimated average oxidation state of each pixel was calculated based on the following linear functions [equations (7)–(10)],

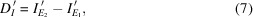




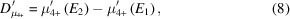




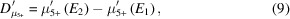




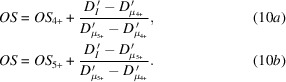

Here, 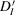 represents the difference between the normalized XRF intensities at *E*_1_ and *E*_2_; 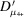 and 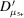 represent the difference between the normalized absorption at *E*_1_ and *E*_2_ of U^4+^ and U^5+^ reference compounds, respectively. *OS* is the estimated average oxidation state of each pixel within the µ-XRF map, while *OS*_4+_ and *OS*_5+_ are the bounding oxidation state values (4 and 5, respectively) of the linear calibration function [equations (10 *a*) and (10 *b*)]. It is worth noting that the oxidation states can be estimated outside of a reasonable range due to the effect of local U absorber concentration. A simple adjustment is required to modify the range by recalibrating the range into a suitable lower and upper bound. In this paper, the method without adjustment is referred to as ‘conventional GLF’ and the method with adjustment is referred to as ‘adjusted GLF’.

#### 2.5.2. Normalized absorption linear regression (NLR) analysis

The assumption made in the application of the aforementioned GLF method is that the difference in normalized µ-XRF intensities between *E*_1_ and *E*_2_ is only dependent on the relative fractions of U^4+^ and U^5+^ present. For an unknown sample, the normalized µ-XRF intensities of each pixel in the µ-XRF maps at these selected energies could be attributed to a summation of the products of the relative fraction of U^4+^ and U^5+^ (*a* and *b*) with their respective normalized absorption (coefficient). However, the contrast in normalized absorption is not directly equal to the contrast in normalized µ-XRF intensities [see equation (3)] unless the sample is dilute or thin, as the absorption from other atoms or other edges far outweighs the absorption from the absorber of interest and, therefore, the over-absorption is minimal and may be neglected. For the case of a highly concentrated or thick sample, the normalized µ-XRF intensity is affected by the concentration, which varies at each pixel of the XRF map. It is necessary to introduce a correction function for the translation from normalized µ-XRF intensity, *I*′, into normalized absorption, μ′, with a correction factor (*c*) [equation (11)],

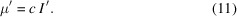

The basic assumption is that the attenuation caused by over-absorption at *E*_1_ and *E*_2_ is identical for the same pixel, and the normalized absorption, μ′, is proportional to the normalized XRF intensity, *I*′. Therefore, the corrected normalized XRF intensity can be expressed by the combination of the relative fraction of U^4+^ and U^5+^ with their respective normalized absorption,

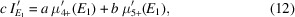




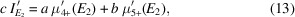




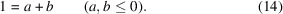

Applying these equations for all collected energy maps, a linear regression model can be written as

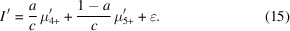

Here, ɛ represents the error using the linear regression method, which is caused by the omitted variables. The values of coefficients *a* and *b* and the correction factor (*c*) can be obtained by using the linear regression [equation (15)] and therefore the average oxidation states can be estimated. It is obvious that the normalized µ-XRF intensities (*I*′), at pixels with higher concentration of absorber, are more attenuated by the over-absorption and require greater correction factors, since the normalized absorption ( 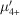 and 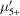 ) of the reference compounds are consistent for all pixels. This linear regression model can be expanded by adding other oxidation states such as U^6+^. However, in this paper, as a pair of energies (*E*_1_ and *E*_2_) were selected for XRF mapping (besides those for normalization and background subtraction) and two reference compounds were used for each oxidation state, only a pair of data were used for the model and, therefore, the linear regression model was effectively a linear function model. It is noted that, without the introduction of *c* and constraints of *a* and *b*, the regression equations could be further simplified to form the expression of conventional GLF method [equation (10)].

#### 2.5.3. Multiple-scattering peak regression (MPR) analysis

The robustness of the normalized absorption linear regression approach can be enhanced by a more accurate estimate of coefficients. Increasing the number of discrete energies at which µ-XRF maps are collected would enlarge the data set for linear regression and so could provide a better prediction. This model could obviously perform better with a large µ-XANES data set from appropriate reference compounds, for training by machine learning (Dyar, Breves *et al.*, 2016 ▸). However, collecting a large number of maps greatly increases data acquisition time and, therefore, statistical methods have been used to reduce the number of energies of interest. Previously, the least absolute shrinkage and selection operator (Lasso) model was applied to reduce the number of discrete energies required for the linear regression expression for predication of Fe oxidation state without a significant decrease in accuracy (McCanta *et al.*, 2019 ▸). Alternatively, deconvolution of the µ-XANES spectrum, using an arctangent and pseudo-Voigt function (as previously described), can also afford more accurate coefficients for the linear regression. As the normalized µ-XRF intensities, *I*′, are not strictly proportional to normalized absorption, μ′ [see equation (16)], a correction factor (*C*) is generally used for XANES over-absorption correction measured in fluorescence mode, based on the compositions of the sample and their corresponding X-ray cross sections according to the FLUO algorithm (Haskel, 1999 ▸),

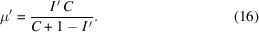

To set up the linear relationship, equation (16) can be rewritten as

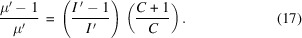

As the coefficients in NLR analysis are assigned to the normalized absorption, the dependence of these coefficients was partially attributed to the edge step which has a non-negligible contribution to the normalized absorption at both *E*_1_ and *E*_2_, which is ignored in the NLR method. Subtracting the edge step from the normalized absorption and using the multiple-scattering peak at *E*_1_ and *E*_2_ augments the independence of the normalized absorptions. 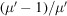 can be considered as the relative normalized absorption over the edge step and 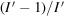 can be interpreted and simplified as the relative multiple-scattering intensity over the edge step of normalized µ-XRF intensities. Therefore, the equations [equations (12)–(14)] representing the relation between normalized absorption and a combination of normalized µ-XRF intensities can be adjusted as

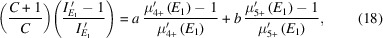




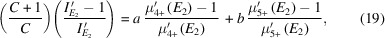




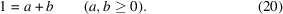

In addition, as the multiple-scattering peak is more sensitive to the local environment, using the multiple-scattering peak provides a better estimate of the correction factor for attenuation caused by over-absorption.

## 3. Results and discussion

### 3.1. Elemental distribution analysis

**Figure 2:**
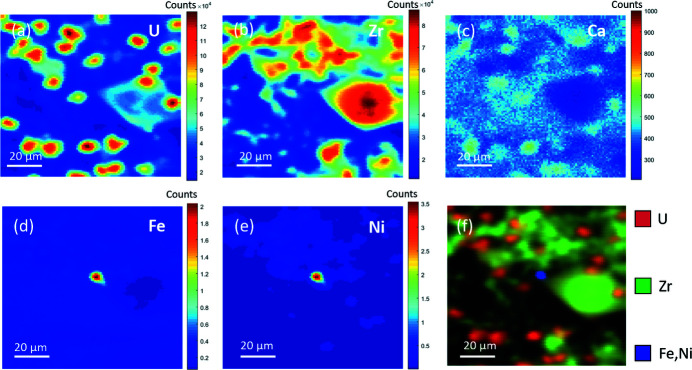
XRF maps of a simulant Black LFCM with an excitation energy of 18000 eV showing the distribution of (*a*) U *L*α, (*b*) Zr *K*α, (*c*) Ca *K*α, (*d*) Fe *K*α, and (*e*) Ni *K*α fluorescence signals; and (*f*) the combined distribution of these elements. The individual map is 150 µm × 150 µm in size, comprising a total of 10000 pixels.

The elemental distributions of U, Zr, Ca, Fe, Ni, and the combined spatial distribution of the elements of interest within a sample of simulant Black LFCM are displayed in Fig. 2 ▸. The µ-XRF maps of the simulant Brown LFCM sample were also determined, and are shown in Figure S1 of the supporting information. The excitation energy was set to 18000 eV, which is sufficient to stimulate emission of Zr *K*α characteristic X-rays. The correlation of U and Zr distributions are in excellent agreement with the previous study of phase assemblages obtained by a combination of µ-XRD and µ-XRF analysis (Ding *et al.*, 2021 ▸). High U and Zr contents were observed in regions where the dominant observed phases included (U,Zr)O_2_ and (U,Zr)SiO_4_. The different crystallite morphologies are also in agreement with previous observations of these materials (Barlow *et al.*, 2020 ▸). Maps of Ca abundances show that it was associated with the glass matrix, where the concentrations of U and Zr were significantly lower, compared with regions dominated by crystalline phases. The distribution of Fe and Ni was concentrated in Fe–Ni alloy particles, as observed in a previous study (Ding *et al.*, 2021 ▸); an example is shown in the approximate centre of the µ-XRF maps shown in Fig. 2 ▸, between the edges of two neighbouring crystallites.

**Figure 3:**
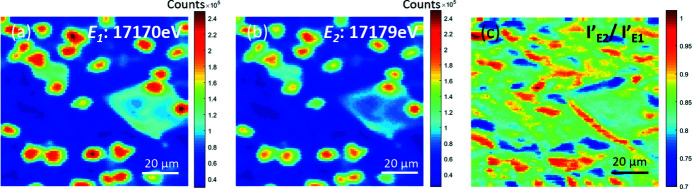
XRF map of Black LFCM at (*a*) *E*_1_: 17170 eV, (*b*) *E*_2_: 17179 eV and (*c*) the normalized XRF intensity ratio 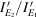 .

The observed U *L*α µ-XRF intensities at *E*_1_ and *E*_2_ have no significant change, as shown in Figs. 3 ▸(*a*) and 3(*b*), demonstrating that the variation of average oxidation states within the material is subtle. Further processing of the data was performed to investigate variation of the average oxidation state change, and so identify regions of interest, by calculating the ratio of normalized µ-XRF intensities at *E*_1_ and *E*_2_ ( 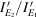 ), as shown as Fig. 3 ▸(*c*). This is an effective method for preliminary observations of the average U oxidation states present, as this ratio is not affected by the normalization process [ 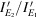 = 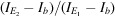 ]. Along the edges of the (U,Zr)SiO_4_ zircon crystallite [large, angular feature seen at the centre-right of Figs. 2 ▸(*b*) and 2 ▸(*f*), and Fig. 3 ▸], the edges of the particles of (U,Zr)O_2_ with a fused morphology, and within the glass matrix, the normalized µ-XRF intensity ratio is higher than those regions in the centre of U-containing features. However, a fraction of the pixels had a lower normalized µ-XRF intensity ratio compared with the normalized µ-XANES absorption ratio of the UO_2_ reference compound ( 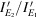 = 0.83). This suggests the different XRF intensities at *E*_1_ and *E*_2_ are caused by the variation of U concentration which is comparable with that arising from average oxidation state.

### 3.2. Chemical state mapping

**Figure 4:**
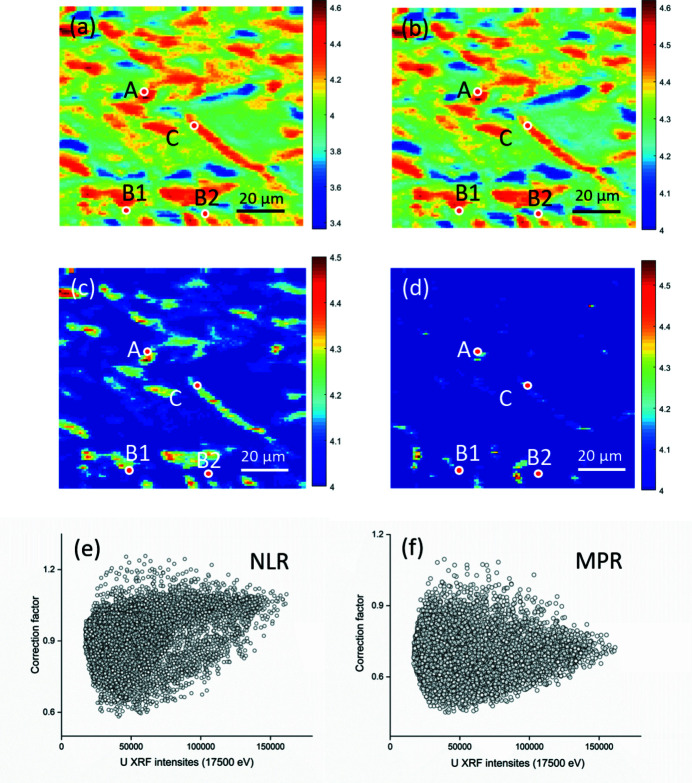
Chemical state map of Black LFCM based on (*a*) linear function analysis (conventional GLF), (*b*) linear function with simple adjustment (adjusted GLF), (*c*) normalized absorption linear regression (NLR) analysis, (*d*) multiple-scattering peak regression (MPR) analysis, (*e*) correction factor of NLR against U XRF intensities at post-edge (17500 eV), and (*f*) correction factor of MPR against U XRF intensities at post-edge (17500 eV).

Maps of the average U oxidation state [see Fig. 4 ▸(*a*)] were constructed from µ-XRF measurements by use of the conventional GLF method discussed above. This chemical state map simply utilized the difference in normalized µ-XRF intensities at *E*_1_ and *E*_2_, affording estimated average U oxidation states of 3.4 to 4.6. To remove the effect of local concentration, the lower bound of the U oxidation state present was set to 4 and the upper bound remained as 4.6. All pixels were recalibrated by reducing the size of the interval between the lowest estimated values and the upper bound, from 1.2 (range of estimated oxidation states 3.4 to 4.6) to 0.6 (range of 4.0 to 4.6), shown in Fig. 4 ▸(*b*). Regions containing higher average U oxidation states (>4.3) were mainly located along the edges of U-bearing particles and between the edges of particles and the glass matrix.

The chemical state map calculated using the normalized absorption linear regression (NLR) method is shown in Fig. 4 ▸(*c*). The spatial distribution of average U oxidation states shows that regions of generally higher and lower oxidation state are correlated with those in the conventional and adjusted GLF analysis. The range of estimated average oxidation states is smaller in the NLR method (4.0 to 4.5) than in the conventional GLF method (3.4 to 4.6) and adjusted GLF method (4.0 to 4.6). There are two main reasons for this improved accuracy. Firstly, the correction factor [*c*, see equations (11) and (12)] enables the differentiation of pixels with the *same* normalized µ-XRF intensity difference at *E*_1_ and *E*_2_ but *different* absorber (U) concentrations. The variation of the correction factor results in differing ratios of U^4+^ and U^5+^, and so the corresponding estimated oxidation states. In these cases, utilizing the conventional GLF method leads to identical values of estimated average oxidation states, as it is only dependent on the normalized µ-XRF intensities. The narrower range of average oxidation states as calculated using the NLR method [see Figs. 4 ▸(*b*) and 4 ▸(*c*)] shows a reduced impact of the concentration effect, as well as more accurate oxidation state estimation. A further advantage of the NLR method is that it eliminates unreasonable estimated average oxidation states (*e.g.* estimated U oxidation states below U^4+^). It constrains the range of oxidation states attributable to the sample by setting lower bounds on the predicted U^4+^ and U^5+^ contents (*a*, *b* ≥ 0). Consequently, a fraction of oxidation states estimated by conventional GLF method are less than U^4+^ due to the effect of local U concentration; whilst the NLR method would correctly predict the material to be composed of U^4+^ only at these pixels.

The chemical state map based on the MPR method is shown in Fig. 4 ▸(*d*). It displays a marginally wider range of oxidation states and contrast compared with the NLR analyses (4.0 to 4.6, compared with 4.0 to 4.5 for the NLR method). This is attributed to a reduction in the dependence on normalized µ-XRF intensities at *E*_1_ and *E*_2_, when compared with the NLR method. Meanwhile, in regions with higher oxidation states (particularly the edges of particles) it eliminates some subtle variation in the estimated average oxidation states, which were likely caused by differences in U concentration rather than actual variations in U oxidation state. These differences in chemical state maps originated from the variation of correction factors for different U concentration [Fig. 4 ▸(*e*) and 4(*f*)]. The correction factors of the NLR method [Fig. 4 ▸(*e*)] tend to increase with U concentration (XRF intensities at post-edge) due to the assumption mentioned above that attenuation caused by over-absorption changes linearly with absorber concentration. The distribution of correction factor of the MPR method is less proportional to U concentration, since the multiple-scattering peak rather than the normalized absorption/intensity is applied for the estimation of correction factor. The use of the multiple-scattering peak as a calibration dataset gave better correction for attenuation. Comparison of the chemical state map derived from the adjusted GLF method [Fig. 4 ▸(*b*)] and that from the MPR method [in Fig. 4 ▸(*d*)] is particularly insightful. In Fig. 4 ▸(*b*), red areas with an apparent average U oxidation state of 4.3–4.6 are revealed to have a true average U oxidation state close to 4.0, validated by point µ-XRD, µ-XANES and µ-EAXFS as discussed below. Clearly, Fig. 4 ▸(*b*) is grossly misleading in terms of communicating the heterogeneity of average U oxidation states present in the material. Indeed, we utilized such a map to select points of interest for further analysis during on-the-fly analysis, erroneously believing that some of these points to be micro-domains characterized by significantly higher average U oxidation states.

**Figure 5:**
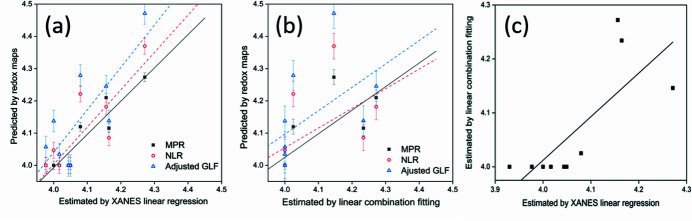
Comparisons of oxidation states calculated using three different methods of analysis of µ-XRF maps and (*a*) µ-XANES linear regression (LR) and (*b*) linear combination fitting (LCF) of µ-XANES spectra. The dotted lines are regression lines for three chemical state maps, respectively. (*c*) Comparison of oxidation states estimated by µ-XANES linear regression (LR) and linear combination fitting (LCF) of µ-XANES spectra.

To confirm the distribution of assigned oxidation states and compare the accuracy of the results obtained by the different methods detailed above, several representative points (*n* = 10) of interest were selected and their µ-XANES spectra acquired (see Fig. S2). A comparison of the oxidation states predicted by the three chemical state mapping techniques, and µ-XANES linear regression (LR) and linear combination fitting (LCF) is shown in Fig. 5 ▸. The MPR analysis of the µ-XRF maps is in better agreement with the µ-XANES spectra (with *R* ^2^ values of 0.93 for LR and 0.77 for LCF, respectively), compared with NLR analysis (*R* ^2^ of 0.84 for LR and 0.52 for LCF) and adjusted GLF analysis (*R* ^2^ of 0.80 for LR and 0.52 for LCF). The deviation between analysis of the µ-XRF maps and analysis of the µ-XANES spectra can be attributed to the different number and selections of feature points applied for estimation [linear regression uses a single inflection point; LCF uses all points of the XANES spectrum; and XRF chemical state maps use two energy points (*E*_1_ and *E*_2_)].

**Figure 6:**
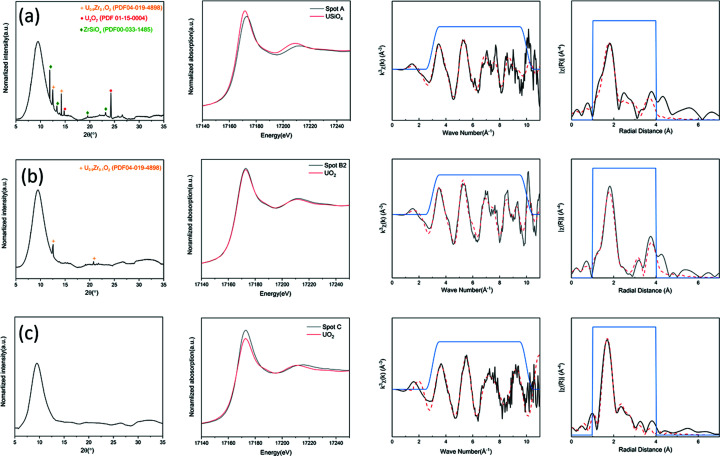
The analysis of µ-XRD patterns, µ-XANES spectra, and µ-EXAFS spectra in radial space and the corresponding *k* ^3^-weighted EXAFS spectra of (*a*) point A, (*b*) point B2 and (*c*) point C.

To verify these observations, µ-XRD patterns and µ-EXAFS spectra were collected at a further set of representative points to obtain information on the phases present and the U speciation [see Fig. 4 ▸(*d*)]. Point A is located at the edge of a particle of (U,Zr)SiO_4_ zircon; B1 and B2 are a pair of points in the region of fused particles generally composed of (U,Zr)O_2_; and point C is within the glass matrix. Figure 6 ▸ shows the analysis of µ-XRD, µ-XANES, µ-EXAFS spectra in radial space and the corresponding *k* ^3^-weighted µ-EXAFS spectra of these points. The oxidation states of these points estimated by different methods, based on analysis of µ-XANES spectra and µ-XRF chemical state maps, are shown in Table 1 ▸.

At point A (located on the edge of a particle of zircon), the reflections identified in the µ-XRD pattern corresponded to (U,Zr)SiO_4_, UO_2_ and U_3_O_7_. The µ-XANES spectrum displayed a shift in energy compared with that of a well characterized U^4+^ coffinite (USiO_4_) reference compound, revealing that a portion of the U was present as oxidized U^5+^ and/or U^6+^ species. The average oxidation state of U within the glass as estimated by the MPR method (4.3) was in excellent agreement with both methods of analysis of µ-XANES spectra, whilst both the adjusted GLF (4.5) and NLR methods (4.4) gave slightly higher estimates. This verified that the difference in normalized µ-XRF intensities at *E*_1_ and *E*_2_ was influenced by both local U concentration and oxidation state. The best fit of the µ-EXAFS spectrum used USiO_4_ as a starting model, and showed a shorter average U—O bond length than the model USiO_4_ (see Table S1), and afforded a bond valence sum of 4.5 v.u., consistent with interpretation of the chemical state map. The bond valence sum value is an empirical estimation of the oxidation state as the oxidation state changes with metal–ligand distances (Kanowitz & Palenik, 1998 ▸). This demonstrates that the mapping approaches developed here are capable of identifying subtle differences in average U oxidation state in complex LFCM materials, enabling both reliable and rapid surveying of the material and identification of points of interest based on accurate estimation of average U oxidation state.

The µ-XRD patterns and µ-XANES and µ-EXAFS spectra of points B1 and B2 are all similar. The reflections identified within the µ-XRD patterns corresponded to cubic (U,Zr)O_2_; the µ-XANES spectra are essentially identical to that of the UO_2_ reference compound; and the fits of the µ-EXAFS spectra show that both exhibited a contraction in the unit-cell parameter compared with UO_2_ (see Table S1), due to the presence of Zr substitution (Zr^4+^ ionic radius = 0.84 Å; U^4+^ ionic radius = 1.00 Å, for eight-fold co-ordination). The average U oxidation state of point B1, as estimated by NLR and MPR methods of µ-XRF map analysis, is 4.0 (4.3 for adjusted GLF), which is in excellent agreement with the oxidation states identified from the two methods of analysis of the µ-XANES spectrum. For point B2, the average U oxidation state estimated using the MPR method (4.0) is identical to that determined by linear regression and linear combination analysis of the µ-XANES spectrum; whilst those calculated utilizing the other methods of µ-XRF map analysis, adjusted GLF and NLR, are slightly higher (4.2 and 4.1, respectively). This deviation in the adjusted GLF and NLR methods can be ascribed to the overestimation of the contribution of the U^5+^ at the lower energy position.

At point C (corresponding to a region within the glass matrix), the µ-XRD pattern consisted only of diffuse scattering from an amorphous phase, and no Bragg reflections were observed. The µ-XANES spectrum of point C shows a shift in *E*_0_ to higher energy compared with that of the coffinite (USiO_4_) reference. Fitting of the µ-EXAFS spectrum (see Table S1) indicated that U is present within the glass in eight-fold coordinated sites (with a bond valence sum of 4.2 v.u), similar to the UO_8_ polyhedra present in crystalline USiO_4_. The oxidation states obtained by the three methods of analysis of the µ-XRF maps are all 4.2, which are in excellent agreement with those estimated from linear regression and LCF of the µ-XANES spectrum (4.2 and 4.3, respectively).

The results of the analysis of the selected points demonstrated that the MPR method of U oxidation state determination from µ-XRF maps are more accurate compared with the adjusted GLF and NLR methods. Using 2D µ-XRD to determine the phases present allows for selection of reference compounds representative of the U speciation in the amorphous phase, and so construction of more accurate maps of the U oxidation state. Multiple-scattering peak regression analysis offers a reliable and accurate approach for the extraction of oxidation states from µ-XRF maps when reference compounds of similar absorber local coordination environment are available. By applying this method, maps of estimated U oxidation state, in complex and heterogeneous materials, can be simply and rapidly constructed.

## 4. Conclusion

In this study, we have characterized simulant Chernobyl LFCMs using micro-focus spectroscopy and diffraction techniques and constructed maps of average U oxidation state using a variety of methods of data analysis. Regions containing higher average U oxidation states were observed to be mainly located along the edges of U-bearing particles and between the edges of particles and the glass matrix. Three different methods of analysis of chemical state maps (gradient linear function, normalized linear regression and multiple-scattering regression) have been compared with two methods of analysis of µ-XANES spectra (linear regression and linear combination fitting), with oxidation states calculated by multiple-scattering regression analysis yielding better accuracy. This was attributed to the corrections of both the local U concentration effect and the effect of over-absorption utilized in the multiple-scattering regression method. Further investigation of representative points by µ-XRD and µ-EXAFS verified the estimated oxidation states by comparison with the phases identified and the changes in their U chemical environment. We have demonstrated that the oxidation states of U can be evaluated accurately by the multiple-scattering regression method, providing a widely accessible method that can simply and rapidly construct maps of U oxidation states.

Previous approaches to chemical state mapping mainly focused on the oxidation state of the elements of interest (Price *et al.*, 2015 ▸; Schroer *et al.*, 2003 ▸). These approaches were based on a single calibration point extracted from µ-XANES spectra of different reference compounds; whilst this method is simple and broadly applicable, the results are necessarily semi-quantitative or qualitative in nature. Several alternative methods were recently developed for quantitative analysis of oxidation states (Lam *et al.*, 2012 ▸; McCanta *et al.*, 2019 ▸). These offer some improved accuracy in the determination of spatial variation in redox gradients; however, accuracy is affected by over-absorption and data normalization. Quick-scanning XAS techniques (Lützenkirchen-Hecht *et al.*, 2001 ▸; Clark *et al.*, 2020 ▸) are nowadays an alternative approach for rapid and accurate quantitative measurement of oxidation state, on a millisecond time scale in fluorescence mode. However, this is currently limited to point measurements for bulk samples (Schroer *et al.*, 2003 ▸) and not suitable for heterogeneous sample on the micrometre length scale, since the spot size is relatively large (for example at SuperXAS, 100 µm × 100 µm). Compared with these previous studies, the methodology developed here enables improved calibration of speciation maps by determination of the normalized X-ray absorption at excitation energies selected to maximize oxidation state contrast, through application of simple algorithms. This enables an improvement of the accuracy and precision in quantification of spatial variation and gradients of oxidation states, and the method is generally applicable. In addition, no sophisticated equipment (such as a quick-scanning monochromator) is needed, so that measurement can be easily performed at any standard X-ray beamline with sufficient flux and focusing capabilities. This development is also an important tool in understanding the behaviour of U in complex, heterogeneous systems. When coupled with the small sample size, this methodology is highly promising for the analysis of highly radioactive, real, nuclear fuel materials present at Chernobyl and Fukushima, and therefore has the potential to aid the ongoing decommissioning of severely damaged nuclear reactors and other degraded nuclear fuels.

## Supplementary Material

Table S1 and Figures S1 and S2. DOI: 10.1107/S1600577521007748/ok5050sup1.pdf

## Figures and Tables

**Table 1:** U oxidation states of representative points estimated from two methods of analysis of µ-XANES spectra, and three methods of analysis of µ-XRF maps

	µ-XANES		Chemical state maps			
Point	Linear regression	LCF	Conventional GLF	Adjusted GLF	NLR	MPR
A	4.3 ± 0.1	4.2 ± 0.1	4.5 ± 0.1	4.5 ± 0.2	4.4 ± 0.1	4.3 ± 0.1
B1	4.0 ± 0.1	4.0 ± 0.1	4.2 ± 0.2	4.3 ± 0.1	4.0 ± 0.1	4.0 ± 0.1
B2	4.0 ± 0.1	4.0 ± 0.1	4.0 ± 0.2	4.2 ± 0.1	4.1 ± 0.1	4.0 ± 0.1
C	4.2 ± 0.1	4.3 ± 0.1	4.3 ± 0.2	4.4 ± 0.1	4.2 ± 0.1	4.2 ± 0.1
