# Supplementary material for: Chemical state mapping of simulant Chernobyl lava-like fuel containing material using micro-focused synchrotron X-ray spectroscopy
Source: J Synchrotron Radiat. 2021 Sep 13;28(Pt 6):1672–83. doi: 10.1107/S1600577521007748 (PMC8570221; doi:10.1107/S1600577521007748)
Supplement: Supplementary file 1 [file s-28-01672-sup1.pdf]

JOURNAL OF SYNCHROTRON RADIATION

Volume 28 (2021)

Supporting information for article:

Chemical state mapping of simulant Chernobyl lava-like fuel containing material using micro-focused synchrotron X-ray spectroscopy

Hao Ding, Malin C. Dixon Wilkins, Lucy M. Mottram, Lewis R. Blackburn, Daniel Grolimund, Ryan Tappero, Sarah L. Nicholas, Shikuan Sun, Claire L. Corkhill and Neil C. Hyatt

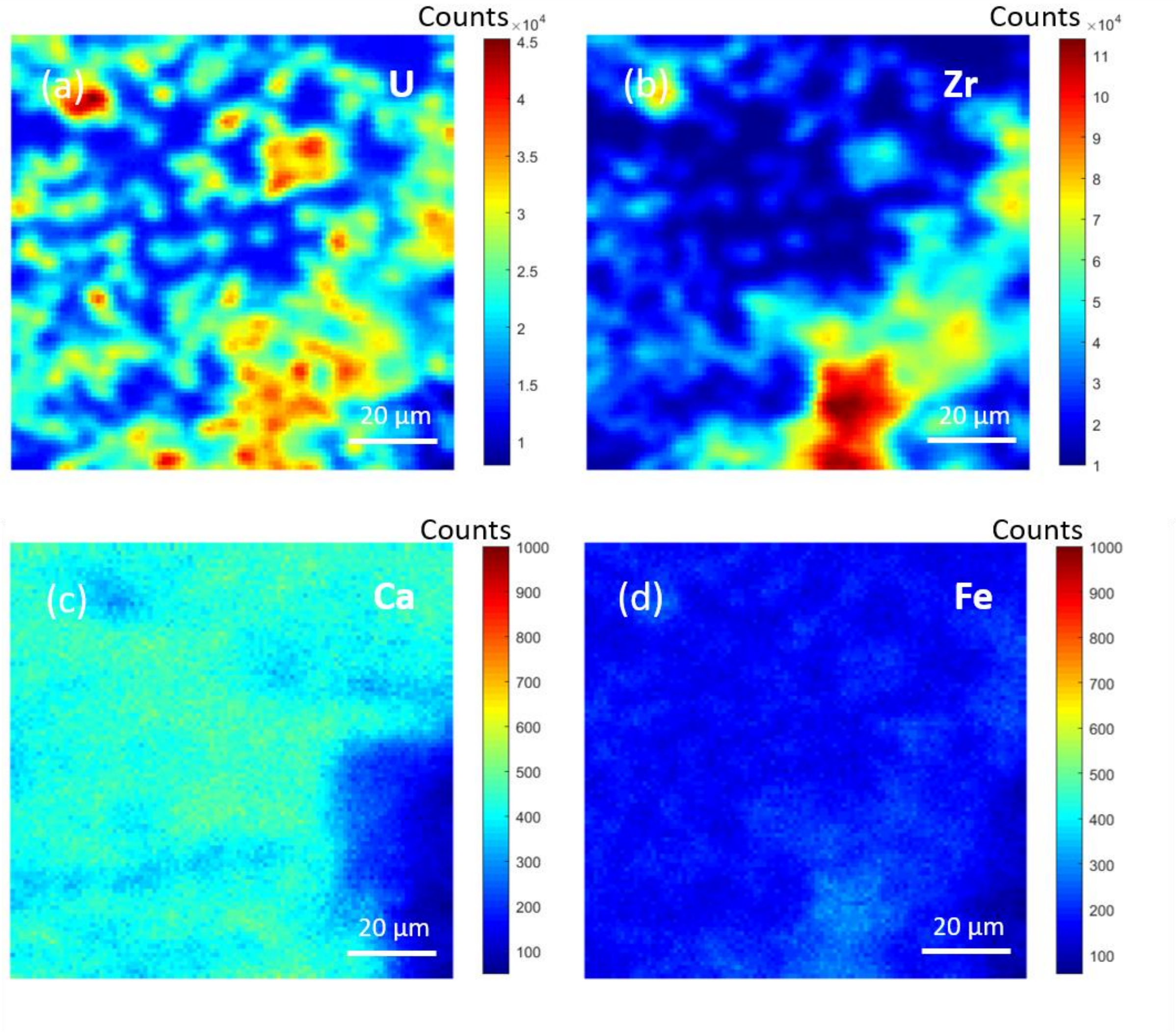

Figure S1 µ-focus fluorescence maps of Brown LFCM at 18,000 eV. Showing distribution of **a)** U Lα, **b)** Zr Kα, **c)** Ca Kα, and **d)** Fe Kα fluorescence signals.

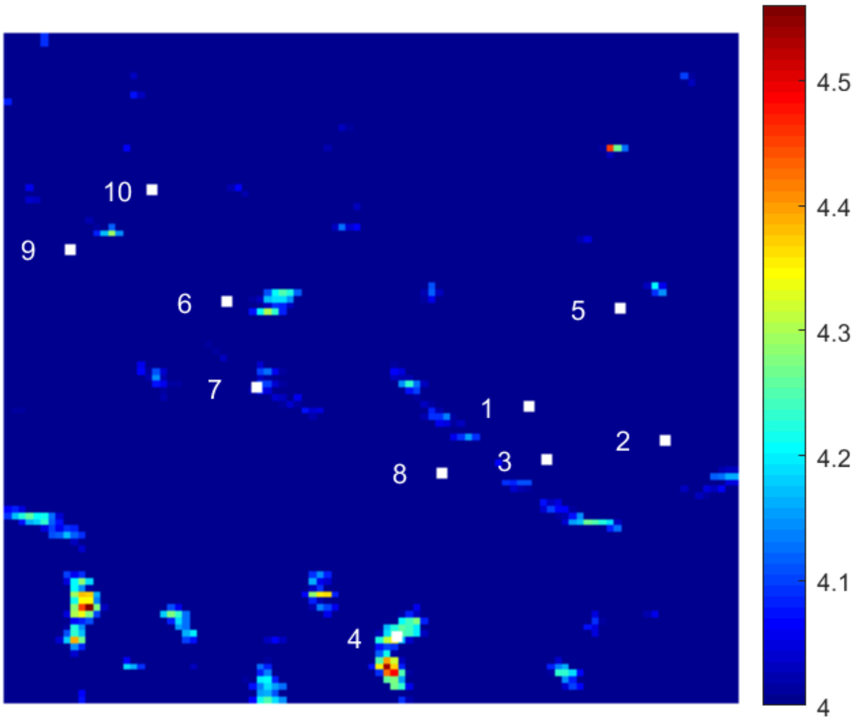

Figure S2 Chemical state mapping of Black LFCM derived from multiple scattering peak regression (MPR) analysis with locations of representative points selected for XANES data acquisition.

Table S1 μ-EXAFS fit parameters of representative points in simulant Black LFCM.

Points	S_0^2	R-factor	N1	U-O(Å)	$\sigma^2(10^{-2}Å^2)$	N2	U-Zr(Si)(Å)	$\sigma^2(10^{-2}Å^2)$	N3	U-U(Å)	$\sigma^2(10^{-2}Å^2)$
A	0.95	0.033	4	2.26 ±0.11	1.04 ± 1.43	4	3.21 ±0.04	0.69 ± 0.57	4	3.81 ±0.05	1.00 ± 0.50
			4	2.36 ±0.13	1.04 ± 1.43	4	3.61 ±0.20	3.57 ± 3.82			
B2	0.95	0.032	8	2.33 ±0.01	1.16 ± 0.10	3.66 ±0.86	3.83 ±0.02	0.91 ± 0.14	8.34 ±0.86	3.83 ±0.02	0.91 ± 0.14
C	0.95	0.039	4.89 ±1.65	2.24 ±0.03	0.31 ± 0.39	2.59 ±2.14	3.15 ±0.03	0.69 ± 0.57			
			2.91 ±0.70	2.42 ±0.05	0.31 ± 0.39						
